# Panton-valentine leukocidin *Staphylococcus aureus* severe infection in an infant: a case report and a review of the literature

**DOI:** 10.1186/s13052-021-01105-5

**Published:** 2021-07-17

**Authors:** Massimo Luca Castellazzi, Samantha Bosis, Irene Borzani, Claudia Tagliabue, Raffaella Pinzani, Paola Marchisio, Giada Maria di Pietro

**Affiliations:** 1 Paediatric Emergency Department, Fondazione IRCCS Ca’ Granda Ospedale Maggiore Policlinico, Milan, Italy; 2 Paediatric Highly Intensive Care Unit, Fondazione IRCCS Ca’ Granda Ospedale Maggiore Policlinico, Milan, Italy; 3 Radiology Unit - Paediatric Division, Fondazione IRCCS Ca’ Granda Ospedale Maggiore Policlinico, Milan, Italy; 4 Department of Pathophysiology and Transplantation, University of Milan, Milan, Italy; 5 University of Milan, Milan, Italy

**Keywords:** Panton-valentine leukocidin, *Staphylococcus aureus*, Children, Infection

## Abstract

**Background:**

Panton-Valentine leukocidin (PVL) is one of the major virulence factor of *Staphylococcus aureus* (SA) that might be associated with invasive life-threating infections. A prompt diagnosis and adequate treatment are essential in achieving the best outcome and avoiding serious sequelae. We describe a case of severe invasive PVL-SA infection in an infant. A literature review starting from 2010 was also performed in order to discuss clinical presentations, radiological findings, treatment and outcome.

**Case presentation:**

This is a case of a 6-month-old boy who rapidly developed high fever and poor general condition. He was diagnosed as having multiple muscular abscesses, multiple foci of osteomyelitis and bloodstream infections caused by Panton-Valentine leukocidin Methicillin-resistant *Staphylococcus aureus*. He received intravenous antibiotics and surgical drainage of the abscess with progressive recovery.

**Conclusion:**

Our report highlights the importance of improving awareness of this severe infection, as a prompt diagnosis and adequate manage is essential in order to save life and to prevent serious complications.

## Background

*Staphylococcus aureus* (SA) is one of the major cause of bacterial infections in humans [1].

Panton-Valentine leukocidin (PVL) is an exotoxin that destroys leukocytes and that is secreted by 2–5% of SA strains [2].

The presence of PVL genes is associated with an increased risk of a more serious infection requiring intensive care, such as necrotising pneumonia, osteomyelitis, septic arthritis, sepsis and multiorgan failure [3].

Due to the potential association with life-threatening and complicated infections, a high suspicion and vigilance for a PVL-positive SA (PLV-SA) infection is essential for a prompt diagnosis and for starting an adequate treatment to achieve the best outcome.

We describe a case of an infant with a severe PLV-SA infection and we perform a literature review since 2010 on this topic in paediatric age.

## Case presentation

A 6 month-old male was admitted to our emergency department for fever since 7 days, erythematous non pruritic skin rash on the trunk, vomiting, diarrhoea and cough.

The child in his first 5 months of life reported four hospitalizations: the first because of an episode of apnoea, the second due to an upper respiratory tract infection, the third and the last one because of two episodes of bronchiolitis. During these hospitalizations, the child performed some immunological investigations which resulted normal and a chest computed tomography (CT) scan that showed a reduced left lower lobe bronchus calibre with accessible segmental and sub-segmental bronchi.

On admission, he was in good general conditions, with normal vital signs, moist mucous membranes, tongue and lips dry, tears when crying, mild tachypnoea (respiratory rate 40/min) and wheezes all over the chest. Laboratory tests showed a C-reactive protein (CRP) of 3.5 mg/dl (normal value < 0.5 mg/dl) and a white blood cell count (WBC) of 11,250/μL, with 22.9% of neutrophils. The stool culture, the research of viruses on faeces and isolation of respiratory viruses on nasopharyngeal aspirate were performed and resulted negative. The patient received inhaled bronchodilator and oral steroid therapy with improving of the respiratory symptoms. The child was hospitalized for further investigations for his history of recurrent respiratory infections.

On day three, he performed a bronchoscopy with a bronchoalveolar lavage (BAL). Four days later, the patient’s clinical conditions worsened, presenting high fever, clinical signs suggestive of sepsis and a swelling of his left shoulder. He was transferred to the paediatric intensive care unit. The blood tests showed an elevation of CRP 65 mg/dl and a WBC of 3800/μL with 55% of neutrophils. A blood culture was also performed. We prescribed a broad spectrum intravenous antibiotic treatment with cefotaxime 100 mg/kg/day. The echocardiography resulted negative. The abdominal ultrasound showed hepatosplenomegaly.

**Fig. 1:**
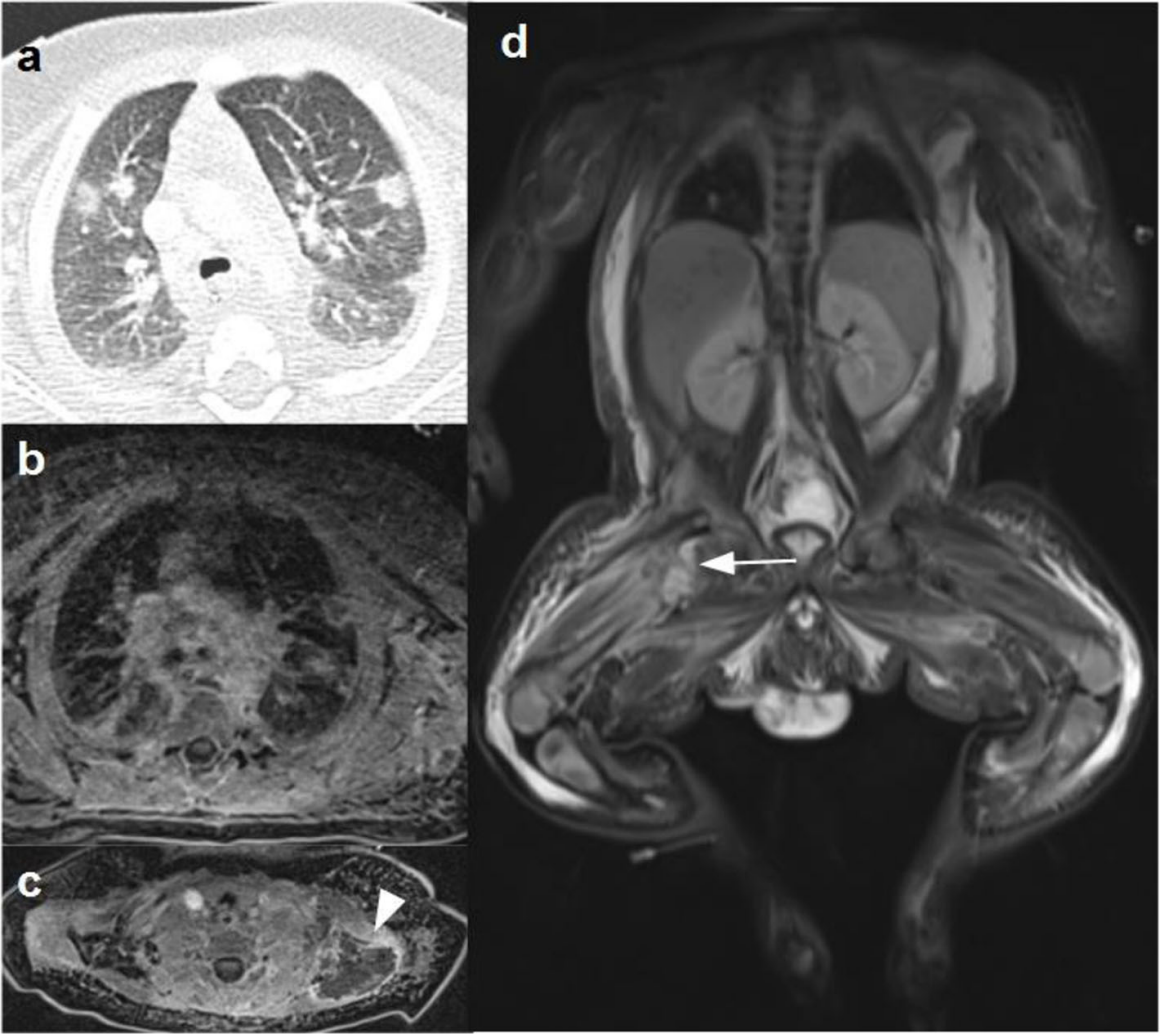
**a**-CT and **b**-Axial T1W-MR image showed scattered lung nodules consisntent with septic emboli. **c**-Axial T1W-FS MR image with contrast medium showed a huge muscular abscess around the left shoulder (arrowhead). **d**- Coronal STIR MR image demonstrated diffuse hyperintense signal of the muscles and the subcutaneous fat throughout the entire body and signal alteration of the right femoral neck associated with right hip effusion (arrow) consistent with osteoarthritis

A contrast-enhanced CT scan of chest and abdomen showed multiple abscesses in the posterior-superior muscles of the shoulder, in the deltoid muscle and in the subscapularis muscle, with pulmonary septic embolisms and fluid collection in the right hip joint. The subsequent whole-body magnetic resonance imaging (WB-MRI) confirmed the flogistic findings of the lungs, demonstrated a wide spread of muscular inflammation in almost all the muscle of the upper part of the body with flogistic collections in the muscle around the shoulder and in the left paravertebral muscles of neck. In addition, osteomyelitis of the proximal metaphyseal region of the right femur and the proximal diaphysis of the left humerus was noted (Fig. 1).

The blood culture resulted positive for a Methicillin-Resistant SA (MRSA), as well the culture of the BAL. The *polymerase chain reaction* revealed that it was a PVL-MRSA, in particular the ST-121 strain. Of note, also *Streptococcus pneumoniae* and *Haemophilus influenzae* were detected in the BAL culture. Therefore, we replaced the antibiotic therapy with a combination of intravenous ceftaroline 24 mg/kg/day, daptomycin 12 mg/kg/day and clindamycin 30 mg/kg/day**.** This antibiotic regimen was continued for 2 weeks and then with only intravenous ceftaroline for others 4 weeks. Five days after the start of the triple antibiotic therapy, the infant underwent a surgical drainage of the extended abscess of the left shoulder, and the culture of the purulent exudate resulted positive for the same PVL-MRSA.

**Fig. 2:**
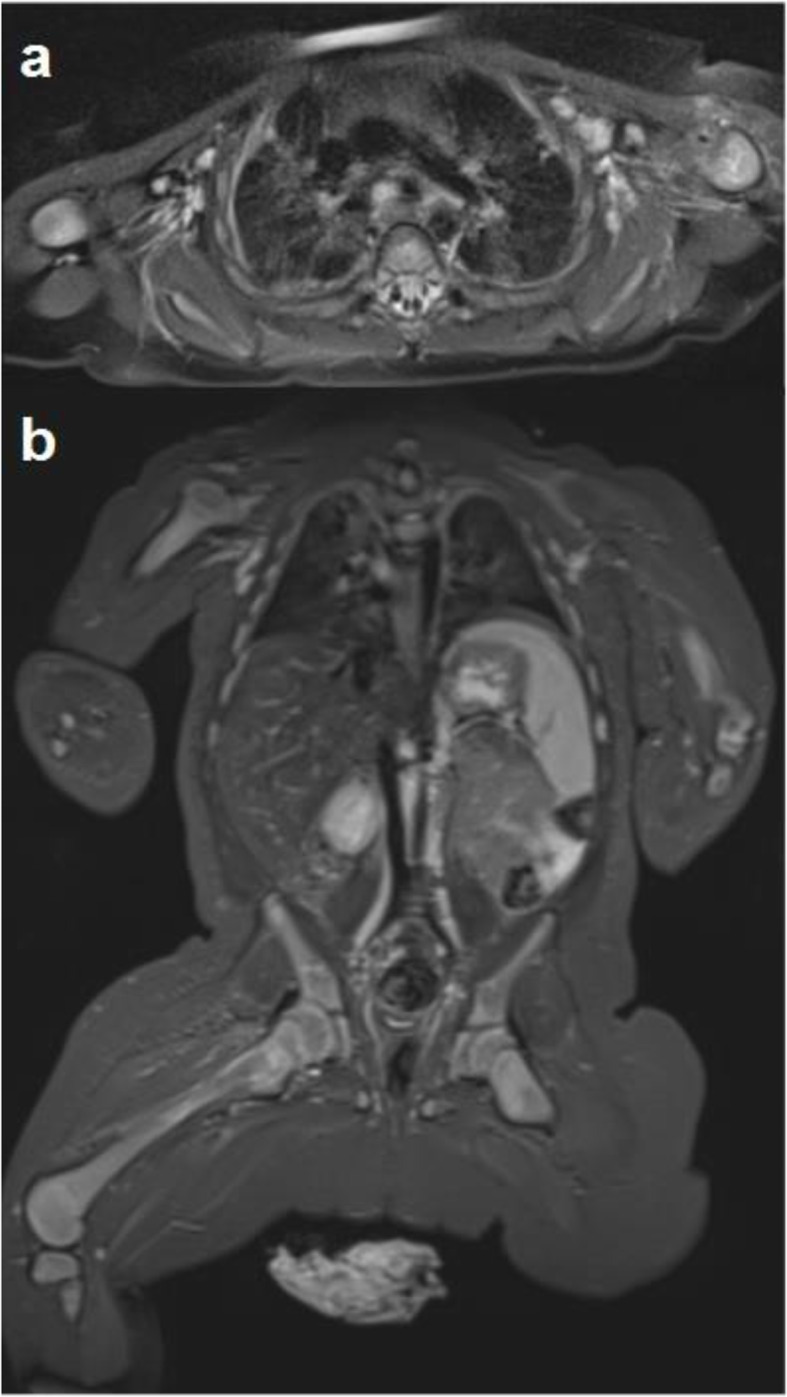
**a**-axial and **b**-coronal STIR MR images performed about 1 month later showed complete resolution of muscular involvement and improvement of lungs and bone flogistic involvement

There was a gradual normalization of the CRP values and an improvement of symptoms and general conditions. Immunological investigations including immunoglobulins and IgG subclasses, lymphocyte subpopulations and tests for complement function (CH50, AP50) resulted normal. The WB-MRI performed 1 month later showed a resolution of the colliquative area in the muscular tissue, an improvement of the bone structure and oedema of the left humerus and the right femur, a reduction of the focal lung lesions with persistence of pleural thickening (Fig. 2).

He was discharged in good general conditions after 7 weeks of hospital stay with oral linezolid 30 mg/kg/day divided in 3 doses for 2 weeks.

In order to eradicate the bacterial colonization, the patient and his close family members received daily bathing with chlorhexidine 2% and intranasal application of mupirocin, because of the positivity of the father’s nasal swab for PVL- MRSA.

The WB-MRI performed 6 months later showed a complete radiological resolution.

## Discussion and conclusion

Here we have described a case of a 6-month-old infant with a severe disseminated PVL-MRSA infection with the aim of increasing attention and knowledge on this type of infection, in which a high suspicious is essential for a prompt diagnosis and to start adequate treatment [4, 5].

PVL is one of the most important virulence factors of SA and it is encoded by *Luk*S-PV and *Luk*F-PV genes [6].

PVL may be produced by different strains of SA, in particular both Methicillin-Sensitive SA (MSSA) and MRSA [7]. In a recent multicentre prospective European study the prevalence of PVL-SA amounted to 18,6 and 7,8% of the isolates were MRSA [8].

Ritz et al. demonstrated that the proportion of PVL-SA is higher in infections caused by MRSA (74–100%) than those caused by MSSA (9–46%). The proportion is dependent on the prevalence of MRSA in the respective regions [9]. The PVL positivity rate is 77–100% in community-associated MRSA infections, while it is less than 4% in hospital-associated MRSA infections [10].

In our patient, the genotype of the PVL-MRSA was ST121, that is, to our knowledge, the first reported in a paediatric patient in Italy. This PVL-SA ST121 strain has been described in literature as mainly disseminated in Africa, Asia and Europe [11].

PVL toxin determines a pore formation in the cytoplasmatic membranes, resulting in leucocyte destruction, tissue necrosis and it contributes to the inhibition of infection clearance by the host immune-system [12]. Therefore, PVL-SA associated infections may be more aggressive and life-threatening. In a retrospective study of Hardy et al., among the six patients with PVL-SA bone and joint infections, there were 2 cases of necrotizing pneumonia, 2 cases of pericarditis and 1 death caused by cardiac tamponade. The microorganism isolated was always a MSSA, due to the low rate of MRSA in the paediatric population of the geographic area where the study was conducted [13]. Recently, in a retrospective study by Hoppe et al. of the 75 children treated for PVL-SA infections, 10 contracted a severe infection including necrotizing pneumonia, necrotizing fasciitis, pyomyositis, mastoiditis with cerebellitis, preorbital cellulitis and recurrent deep furuncolosis in an immunosuppressed patient; MRSA was detected in 4 cases [14].

Since 2010, we have identified 15 reported cases of paediatric PVL-SA severe infections and Table 1 summarizes the main results of these reports regarding the clinical presentations, radiological findings, treatment and outcome [2–5, 15–23].

**Table 1:** Summary of the reported cases of severe PLV *S. aureus* infections in children from 2010 to 2020

Author, Year, Nation, Reference	Patients	Age, sex	Clinical presentation	Radiological findings	Antibiotic treatment	MSSA/MRSA and genotype	Treatment antibiotic duration	Other treatment	Outcome
Esteves, 2010, Portugal [15]	1	14-years, M	Fever, cough, thoracic and right inguinal pain 10 days after a toe trauma	Bilateral pneumonia and minor pleural effusion (RX). Deep venous thrombosis of right femoral vein (ultrasound). Bilateral thoracic infiltrates and pleural effusion (CT). Right hip arthritis (CT)	Intravenous piperacillin-tazobactam followed by intravenous flucloxacillin	MSSA genotype not clarified	4 weeks of intravenous flucloxacillin	Low-molecular weight heparin	Complete resolution
Erturan, 2012, United Kingdom [16]	1	16-years, M	Lethargy, loss of appetite, worsening pain of right knee. Fever. Subsequent multilobar pneumonia and pericardial effusion, septicaemia	Right proximal tibial osteomyelitis (MRI)	Intravenous fluoxacillin, which was substituted on day 9 by intravenous linezolid and daptomycin on day 11 and oral clindamycin	MSSA genotype not clarified	Intravenous linezolid 21 days, intravenous daptomycin 19 days, clindamycin 30 days (which was continued at home)	Arthroscopic washout	Complete resolution
Wangjirapan, 2012, Thailand [4]	1	8-years, M	Fever, low back pain, dyspnoea, abdomen distension with guarding and moderate tenderness, endocarditis	Paravertebral abscess, multiple consolidations in the bilateral basal lungs, pleural effusion, thrombosis inferior vena cava, left common iliac vein, proximal left internal iliac vein, small liver abscess, osteomyelitis 5th lumbar vertebral body (CT).	Intravenous cloxacillin, clindamycin and gentamicin.	MSSA genotype not clarified	Gentamicin 14 days, cloxacilin and clindamycin 6 weeks.	Proper surgical drainages	A CT-scan at follow up demonstrated a significantly decreased lung abscess and left pleural effusion. He ambulated well and went back to school
Ceroni, 2012, Switzerland [2]	1	12-years, F	Fever, swollen left ankle. Redness of left foot.	Large osteomyelitis of left tibia with intramedullary abscesses and significant edema of surrounding muscles (MRI)	Intravenous flucloxacillin and gentamicin	MRSA genotype not clarified	Four weeks of intravenous antibiotics, follow by 4 weeks of oral antibiotic (not clarified)	Recurrent surgical drainage (5 times)	Sever restriction of the motion the left ankle
Haider, 2013, United Kingdom [3]	1	12-years, M	Sore throat, fever, haemoptysis, progressive respiratory failure	Extensive bilateral alveolar pulmonary shadowing (chest X-ray)	Intravenous cefuroxime	Not clarified if MSSA or MRSA	Not clarified	No surgical treatment	Death
Fitzgerald, 2013, United Kingdom [17]	1	14-years, M	Fever, back pain (after rugby training), follow by severe necrotizing pneumonia	Discitis and peridural abscess at L3-L4 (MRI). Bilateral interstitial infiltrates (chest X-ray)	Intravenous flucloxacillin and clindamycin, followed by ceftriaxone, clindamycin, clarithromycin and linezolid	MSSA genotype not clarified	After 7 days he was treated with intravenous ceftriaxone and oral clindamycin for 3 months	No surgical treatment. Intravenous immunoglobulin	Complete resolution
Montagnani, 2013, Italy [18]	3	3-months, M	Respiratory distress and poor feeding	Complete atelectasis of left lung, pyopneumothorax and necrotic areas of parenchyma (CT).	Intravenous vancomycin and clindamycin followed by oral linezolid	MRSA ST30	3 weeks	Chest drainage	Complete resolution
		2-months, M	Subcutaneous abscess in the left perimalleolar area	No bone involvement on ankle MRI	Intravenous teicoplanin and clindamycin	MSSA ST1572	3 weeks	Surgical drainage	Complete resolution
		2-months, F	Fever, coryza, poor feeding, followed by respiratory distress, drooling, bilateral laterocervical swelling and neck stiffness	Retropharingeal abscess	Intravenous vancomycin and clindamycin, followed by intravenous linezolid and then oral linezolid	MSSA ST576	4 weeks	Surgical drainage	Complete resolution
Karli A, 2015, Turkey [19]	1	13-years, M	Right hip pain, fever	Increase signal in the right iliac crest and increased fluid in the right hip joint. Multiloculated abscess in the gluteal region (MRI). Necrotizing pneumonia (CT)	Intravenous cefazolin and clindamycin followed by intravenous linezolid and clindamycin and then oral amoxicillin-clavulanate.	MSSA genotype not clarified.	38 days	Surgical drainage	Complete resolution
Karli A, 2016, Turkey [20]	1	12-years, M	Fever, respiratory distress and hip pain. Poor general condition, somnolence.	Multiple peripherally localized cavitary round lesions in both lungs (CT). Left psoas muscle abscess and left femoral trochanter osteomyelitis (MRI)	Intravenous vancomycin followed by intravenous linezolid and then oral clindamycin	MSSA genotype not clarified.	21 days of intravenous antibiotic treatment. Not clirifed the duration of oral antibiotic treatment.	Surgical drainage	Complete resolution
Hardgrib, 2016, Denmark [5]	1	13-years, M	Fever, left-side knee pain. Follow by sepsis and respiratory distress	Synovitis with knee joint effusion and signs of medullary osteomyelitis in the proximal tibia (MRI).	Intravenous vancomycin and oral linezolid	MRSA ST30	7 weeks	Multiple surgical drainage	Complete resolution
Ravishankar, 2016, USA [21]	1	10-years, F	Emesis that progressed to respiratory distress, pallor and perioral cyanosis, and near syncope upon standing	Not performed	Intravenous vancomycin and ceftriaxone	MRSA genotype not clarified	Not applicable	None	Death
Irenji, 2018, United Kingdom [22]	1	13-years, M	Severe right-side groin pain irradiated to his right leg and lower abdomen. Poor general condition, vomiting	Iliac muscle abscess (ultrasound) Abscesses anterior and posterior to the iliac crest (MRI) Bilateral consolidation within the lung fields (CT)	Intravenous flucloxacillin and cefotaxime followed by intravenous linezolid and clindamycin.	MSSA genotype not clarified.	Not clarified	Multiple surgical intervention	Complete resolution
Ogata, 2019, Japan [23]	1	12-years, F	Fever, lower back pain, followed by bladder dysfunction	Panniculitis of the left retroperitoneal region, swelling of the left ileopsoas, and multiple vein thrombosis of the inferior vena cava (CT). Hyperintensity around the left ileopsoas (MRI). L4 osteomyelitis and abscess near L3 (MRI)	Intravenous ampicillin and cefotaxime. Followed by intravenous vancomycin subsequently associated with cefazolin and clindamycin. Clindamycin was then substituted with rifampicin.	MSSA ST2149	Cefazolin for 5 weeks.	Surgical intervention for the abscess near L3	Complete resolution

Among these reports, only Montagnani et al. described 3 cases of severe PVL-SA infection in infants [18]. We have also described the case of severe PVL-SA infection in a 6 month-old infant. Therefore, even though life-threating infections due to PVL-SA usually occur in older children, we suggest to consider this pathogen also in infants, especially in those with more severe clinical presentation. Furthermore, PVL-SA infection should be suspected in previously healthy young patients with a history of necrotizing skin and soft tissue infections, recurrent abscesses or household cluster infections. Furthermore, it should be suspected in patients with severe musculoskeletal infections and rapidly progressive pneumonia which is usually preceded by a “flu-like” syndrome and in those children with high fever, leukopenia and increased inflammatory markers [18].

PVL-SA strains can be identified by detection of genes encoding PVL or the toxin itself [9]. Conventional bacterial cultures does not differentiate SA producing or not producing PVL [24].

In our patient WB-MRI was really helpful for the detection and location of multifocal flogistic processes and was a valid and sensitive tool during the follow-up period, without radiation exposure of the child. Whole-body MRI is a fast and accurate modality for detection and monitoring of disease throughout the entire body with a variety of applications in the paediatric patient population [25].

Once diagnosed, PVL-SA infection must be treated appropriately, without delay. Initial empirical coverage generally includes an anti-staphylococcal regimen (amoxicillin-clavulanic acid or flucloxacillin when MSSA is suspected and vancomycin when MRSA is suspected) and, an anti-toxic agent able to block the production of the toxin (clindamycin, rifampicin, linezolid, or gentamicin) [26, 27].

In our case report, the child had a life-threatening infection, community-acquired, in a country with a prevalence of MRSA greater than 10%. We chose ceftaroline as anti-MRSA agent as it is effective and safe similarly to vancomycin [28, 29]. Furthermore, concentration below the Minimum Inhibitory Concentration of β-lactams and, to a lesser extent, vancomycin have been shown to enhance PVL secretion, determining more aggressive symptoms [7, 30]. We associated clindamycin, as inhibitor of PVL production. Moreover, in PICU, daptomycin was included in the regimen for the bacteraemia and the osteo-articular infection. This antibiotic regimen was well tolerated by the patient and without adverse reaction. In a recent retrospective study of Syrogiannopoulos et al. it was observed that in children with complicated staphylococcal infections, daptomycin administration alone or in combination with other antimicrobial agents was efficacious and well tolerated [31]. Furthermore, daptomycin was successfully used in 14-year-old boy with tibial osteomyelitis, multilobar pneumonia, pericardial effusion, and septicaemia [16]. In our case, it was an off label use of this antimicrobial agent, introduced due to the severity of the infection.

Generally, a long duration antibiotic therapy is usually needed, in particular in complicated cases [17]. Unfortunately, antibiotic treatment alone is often insufficient, as antibiotic distribution is reduced in necrotic tissue. Surgery plays a major role in achieving a good outcome, with early and complete removal of PVL suppuration from the body and debridement of necrotic tissues. Furthermore, patients with bone and joint infections require more than one surgical procedure [26]. Patients with PVL positive severe infections needed a prolonged hospital stay and a longer recovery times [5].

Decolonisation is part of the process to completely eradicate bacterial colonisation and should involve all household members. Although the optimal eradication strategy for PVL-SA is not known, it is likely to be used the same for MRSA. It includes nasal mupirocin 2% ointment and chlorhexidine 4% body wash [27, 32]. In our case, the origin of the infection is not clear, as previous hospitalizations maybe a risk factor. Also his father may have been the source of the infection, as he was an asymptomatic carrier. Surely, the decolonisation therapy, extended to all the family members, brought to the complete eradication of the bacterial colonisation.

In conclusion, this case highlights the importance of considering PLV-SA as the etiologic agent of life-threatening infection in children. A prompt identification of the toxin is essential in order to start an adequate treatment, that is based not only on prolonged antibiotic treatment but also on surgical drainage in case of abscess or musculoskeletal infection.

## Data Availability

Data sharing was not applicable to this case report because no datasets were generated or analysed during the study.

## Abbreviations

SA: *Staphylococcus aureus*
PVL: Panton-Valentine leukocidin
PLV-SA: Panton-Valentine leukocidin-positive *Staphylococcus aureus*
CT: computed tomography
CRP: C-reactive protein
WBC: white blood cell
BAL: bronchoalveolar lavage
MRSA: Methicillin-Resistant *Staphylococcus aureus*
MSSA: Methicillin-Sensitive *Staphylococcus aureus*
WB-MRI: Whole Body Magnetic Resonance Imaging
